# Impact of using artemisinin-based combination therapy (ACT) in the treatment of uncomplicated malaria from *Plasmodium falciparum* in a non-endemic zone

**DOI:** 10.1186/s12936-016-1408-1

**Published:** 2016-07-02

**Authors:** Joaquín Pousibet-Puerto, Joaquín Salas-Coronas, Alicia Sánchez-Crespo, M. Angustias Molina-Arrebola, Manuel J. Soriano-Pérez, M. José Giménez-López, José Vázquez-Villegas, M. Teresa Cabezas-Fernández

**Affiliations:** Tropical Medicine Unit, Hospital de Poniente, El Ejido, Almería, Spain; Center for the Study of Migration and Intercultural Relations (CEMyRI), University of Almeria, Almería, Spain; Haematology Unit, Hospital de Poniente, El Ejido, Almería, Spain; Tropical Medicine Unit, Distrito Poniente, Almería, Spain

**Keywords:** Malaria, Uncomplicated malaria, *Plasmodium falciparum*, Artemisinin-based combination therapy, Artemisinin, Dihydroartemisinin–piperaquine

## Abstract

**Background:**

Artemisinin-based combination therapy (ACT) has been adopted by the World Health Organization as a first-line treatment for uncomplicated *Plasmodium falciparum* malaria. In endemic regions, it has proven more effective in treating the disease, and even in reducing its transmission. Nonetheless, there is a scarcity of studies carried out in non-endemic areas on imported uncomplicated malaria.

**Methods:**

This is a retrospective, observational study performed on patients diagnosed and admitted with uncomplicated *P. falciparum* malaria between 2004 and 2015. The objective was to compare the parasite clearance period and the average hospital length of stay for patients treated with ACT vs those receiving other treatment regimens.

**Results:**

Eighty-five patients were included in the study. Fifty-one received ACT treatment (dihydroartemisinin–piperaquine) and thirty-four patients were treated with quinine sulfate+doxycycline or atovaquone/proguanil. The parasite clearance period was shorter in the group of patients treated with ACT compared to those receiving other treatment types: 24 h (IQR 24) vs 48 h (IQR 48), p < 0.01. The average hospital stay was also shorter in the ACT group with respect to the second group: 2.67 days (IQR 1.08) vs 3.96 days (IQR 2.87), p < 0.001. A mild case of hepatitis was registered in the group treated with ACT.

**Conclusions:**

ACT treatment of admitted hospital patients with imported uncomplicated malaria from *P. falciparum* reduced the days spent hospitalized as well as producing a more rapid parasite clearance compared to classic treatment. In spite of being treated with safe medications, one has to be alert to possible adverse effects such as hepatitis and delayed haemolytic anaemia.

**Electronic supplementary material:**

The online version of this article (doi:10.1186/s12936-016-1408-1) contains supplementary material, which is available to authorized users.

## Background

Malaria is the most important parasitic disease worldwide. It has been calculated that each year is it responsible for new episodes in around 214 million patients, causing the death of approximately 438,000 the majority of which are children under 5 years of age and pregnant women [1, 2], not to mention incalculable economic loss in endemic countries. In Europe, apart from isolated cases in Greece, malaria continues to be exclusively imported, with approximately 11,000 cases reported each year, making it the most important tropical disease on this continent [3–6]. Most imported malaria cases (as many as 88–98 %) are diagnosed in recently-arrived immigrants or resident immigrants who return to their country of origin to visit friends or family visiting friends and relatives (VFR); sub-Saharan Africa being the region from which most cases originate. Eighty percent of declared imported cases of malaria in Europe are *P. falciparum* [3–5].

Malaria treatment has changed substantially over recent years [7]. Artesunate has proven to be superior to quinine in most situations where complicated malaria is treated [8–12]. In uncomplicated malaria, regimens known as artemisinin-based combination therapy (ACT) demonstrates greater efficacy in treating the disease, reducing transmission in endemic areas and producing lower levels of reinfection [13–17]. For this reason, artemisinin derivatives are currently recommended as first-line medication for *P. falciparum* malaria treatment, whether complicated (severe) or uncomplicated, both in endemic and non-endemic countries [18–20].

Artemisinin treatment leads to a reduction in average hospital stays, more rapid parasite clearance in comparison with any other anti-malarial [11, 12, 17]; and, in complicated malaria, a lowering of the global mortality rate [8, 9]. The majority of these studies have been carried out in endemic areas. In non-endemic countries, in patients with complicated malaria, treatment with artesunate additionally reduced the days spent in intensive care units (ICU) [11, 12]. However, in patients with uncomplicated malaria from *P. falciparum,* there are few studies comparing the differences in non-endemic regions [17].

This study aims to analyse the impact that the use of ACT has had compared to two classic treatments (quinine sulfate and doxycycline/clindamycin or atovaquone-proguanil) to treat patients admitted with uncomplicated malaria from *P. falciparum* to a hospital in a non-endemic area.

## Methods

A retrospective observational study was carried out on all patients admitted with malaria to Hospital Poniente (El Ejido, Almería. Spain) from January 2004 to December 2015. The hospital serves a population of approximately 250,000 people, in an area with a large African immigrant population, many of whom work in horticultural greenhouses. The study included patients over 14 years of age whose reason for hospital admission was exclusively the diagnosis of uncomplicated malaria from *P. falciparum* [19]. Pregnant women and HIV patients were excluded from the study. The disease was diagnosed using direct thin and thick smear microscopy in tandem with a rapid diagnostic test (SD Bioline malaria Ag Pf/Pan^®^) using lateral flow assays. The malarial PCR (semi-nested multiplex PCR) was used only in cases where a more precise confirmation was needed of the species, or where there was a suspicion of mixed parasitic infection. A clinical history and complete physical examination were carried out on all patients, gathering epidemiological and clinical data. On admission, screening was performed for the most prevalent imported pathologies; this included HIV, HBV and HCV blood tests. Parasitaemia monitoring was carried out via a daily smear until a negative result was obtained. After leaving hospital, the patients were checked with a blood test and a blood smear at 7 and 28 days.

Uncomplicated malaria is defined following the recommendations for the management of imported malaria in Europe [19]. With regard to parasitaemia, patients who came from endemic countries (semi-immune patients) were considered uncomplicated if they had a parasitaemia level of <5 and <2 % in non-immune individuals. Dihydroartemisinin–piperaquine (Eurartesim^®^ 320 mg/40 mg, Sigma Tau) was the ACT chosen; this medication is currently the commercial form available in Spain.

Variables were analysed corresponding to epidemiological, clinical and analytical characteristics as well as the parasite clearance period and the average hospital stay. The level of parasitaemia was divided into patients with <1 %, between 1 and 2 %, and >2–5 %. Any possible adverse secondary effects associated with the treatment were also recorded.

Data were analysed using the statistical software package SPSS v17. A descriptive analysis was carried out on the quantitative variables using the mean and the standard deviation, as well as the range. Qualitative variables were described using absolute frequencies and percentages. To observe the possible differences between the group of patients treated with quinine and doxycycline/clindamycin or atovaquone–proguanil and ACT, the Pearson’s Chi square test or Fisher’s exact test was used when the variables were qualitative. When the variables for comparison were quantitative, the mean differences were estimated using the Student’s t test for independent samples if they complied with the normal hypothesis and the Mann–Whitney U test when they did not.

A multivariate linear regression model was performed using days of hospitalization as the dependent variable and the treatment group, the haemoglobin (Hb) and platelet levels (at the time of diagnosis) and the level of parasitaemia as the independent variables. The goodness-of-fit was calculated along with the determination coefficient. For all tests, the fixed level of significance was 0.05. This study was approved by the local ethic committee (Almería, Spain) with code 12/2016.

## Results

A total of 85 patients diagnosed with uncomplicated malaria were included in the study. The pharmaceuticals used for malaria treatment were quinine sulfate+doxycycline or atovaquone/proguanil until 2012. In the same year, dihydroartemisinin–piperaquine was introduced, remaining as the first-line treatment since then. Fifty-one patients were treated with dihydroartemisinin–piperaquine and 34 with quinine sulfate+doxycycline (n = 33) or atovaquone/proguanil (n = 1). The dataset supporting the conclusions of this study is included as Additional file 1.

The general patient characteristics, both together and by groups, are shown in Table 1. The average age was 32 years (IQR 9), 96.5 % of which were male (n = 82). All of the patients except one (a Spanish expatriate) originated from sub-Saharan Africa, and 94.1 % were treated as VFR patients. No differences were observed on admittance between the study groups with relation to age, gender, country of origin, type of traveler, or basal Hb and platelet levels, nor the level of parasitaemia. Regarding concomitant diseases, one patient was diabetic (in the quinine sulfate+doxycycline group), two presented with arterial hypertension (in the quinine sulfate+doxycycline group), and ten patients presented with chronic hepatitis B (five in the quinine sulfate+doxycycline group and five in the dihydroartemisinin–piperaquine group).

**Table 1:** Characteristics of the patients included in the study

	All N = 85	Quinine/atovaquone–proguanil n = 34	Dihydroartemisinin–piperaquine n = 51	p value
Age (years); median				
(IQR)	32 (9)	31.5 (8)	33 (10)	0.99
Gender				
Male	82 (96.5 %)	32 (94.1 %)	50 (98 %)	0.56
Female	3 (3.5 %)	2 (5.9 %)	1 (2 %)	
Origin				
Sub-Saharan Africa	84 (98.8 %)	34 (100 %)	50 (98 %)	0.49
Spain	1 (1.2 %)	0	1 (2 %)	
Type of traveller				
VFR	80 (94.1 %)	31 (91.2 %)	49 (96 %)	0.25
Recent arrival	4 (4.7 %)	3 (8.8 %)	1 (2 %)	
Expatriate	1 (1.2 %)	0	1 (2 %)	
Hb on admission (g/dL) ± typical deviation (range)	13.7 ± 1.5 (10.1–16.9)	13.5 ± 1.5 (10.1–16.9)	13.8 ± 1.4 (10.1–16.4)	0.36
Hb day 7 (g/dL) ± typical deviation (range)	13.08 ± 1.49 (10–16)	12.65 ± 1.42 (10–15.5)	13.37 ± 1.48 (10–16)	0.03
Hb day 28 (g/dL) ± typical deviation (range)	13.94 ± 1.34 (9.1–16.8)	13.45 ± 1.38 (9.1–14.8)	14.32 ± 1.2 (11.7–16.8)	0.02
Total platelets ×10^3^/µL; median (IQR)	94 (60)	93 (59)	95 (81)	0.93
Parasitaemia level (%)				
<1	48 (56.5 %)	18 (52.9 %)	30 (58.8 %)	0.22
1–2	26 (30.6 %)	9 (26.5 %)	17 (33.3 %)	
>2–5	11 (12.9 %)	7 (20.6 %)	4 (7.8 %)	
Time in which parasitaemia becomes negative. Hours; median (IQR)^a^	24 (24)	48 (48)	24 (24)	<0.01
Period of hospitalization. Days; median (IQR)	2.88 (1.88)	3.96 (2.87)	2.67 (1.08)	<0.001

^a^In the group of patients treated with quinine sulphate, the average parasite clearance time was determined in 16 patients. In the remainder (n = 18) daily smears were not carried out. The variables gender, origin, type of traveller, haemoglobin (basal levels, 7 and 28 days) and parasitaemia level follow a normal distribution. The quantitative variables that do not follow a normal distribution pattern are age, platelets, length of hospital stay and parasite clearance time

**Fig. 1:**
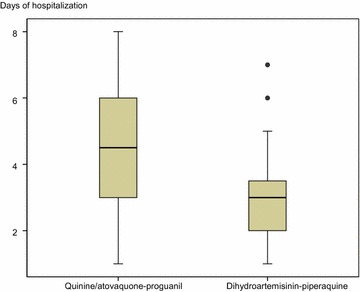
Days of hospitalization per treatment group

The global parasite clearance time was 24 h (IQR 24) and the average hospital stay was 2.88 days (IQR 1.88). In the subgroup analysis, the parasite clearance time was significantly less in the patient group treated with ACT rather than the classic treatment: 24 h (IQR 24) vs 48 h (IQR 48), p < 0.01. The average stay was also less in the artemisinin group with respect to the group treated with quinine+doxycycline/atovaquone-proguanil: 2.67 days (IQR 1.08) vs 3.96 days (IQR 2.87), p < 0.001 (Table 1; Fig. 1).

With regard to the Hb levels during the follow-up at 7 and 28 days, patients treated with dihydroartemisinin–piperaquine presented higher levels on both occasions (ACT group vs quinine+doxycycline/atovaquone-proguanil: day 7: 13.37 g/dL ± 1.48 (range 10–16) vs 12.65 g/dL ± 1.42 (range 10–15.5), p = 0.03; day 28: 14.32 g/dL ± 1.2 (range 11.7–16.8) vs 13.45 g/dL ± 1.38 (range 9.1–14.8), p = 0.02.

In the lineal regression model (Table 2), the variables showing an influence on the average days of hospitalization were the Hb and platelet levels at the time of admission and whether there was elevated parasitaemia (>2–5 %). The variable with the greatest association power was the treatment group, with a typified coefficient of −0.409. For the same Hb, platelet and parasitaemia levels, the use of ACT resulted in a reduction in hospital stay of almost a day and a half (−1.35 days).

**Table 2:** Linear regression model

	Coefficient		Typified coefficient	
	B	Typical error	Beta	Significance
Treatment with quinine/atovaquone–proguanil or ACT	−1.349	0.293	−0.409	<0.001
Hb on admission	0.314	0.097	0.284	<0.01
Platelets on admission	−0.007	0.002	−0.258	<0.01
Average parasitaemia	−0.009	0.319	−0.003	0.98
High parasitaemia	1.083	0.449	0.225	0.02

*ACT* artemisinin-based combination therapy

R^2^ = 0.404 (p < 0.001). The model we have constructed explains 40.4 % of the variability in the average hospital stay. The variables that influence the average stay are the type of treatment, Hb, platelets and having elevated parasitaemia. The variable with the greatest power of association is the treatment type, which has a typified coefficient of 0.409. For the same level of Hb, platelets or parasitaemia, the use of ACT results in almost 1 day less of hospitalization (−1.35 days)

With regard to any reported adverse effects, in the group treated with dihydroartemisinin–piperaquine, there was one mild case of hepatitis, in the only non-sub-Saharan patient; this was self-limiting but, nevertheless, led to more days spent in hospital (6 days).

## Discussion

ACT is a very effective and safe treatment in the management of imported uncomplicated malaria. Besides producing more rapid parasite clearance, its use in patients admitted with uncomplicated malaria reduces the period of hospitalization compared to classic treatments that were used previously, resulting in healthcare savings.

The management of imported malaria is still a challenge for many healthcare professionals in non-endemic zones. *P. falciparum* is the species most frequently involved, and also the one which most results in morbidity and/or mortality in travellers (tourists and expatriates), as well as in foreigners who return home to visit friends and family [3–5]. The use of artemisinin derivatives has been spreading, various articles have been published showing the superior results of these drugs compared to other treatments; indeed, their use has been recommended as a first-line drug in the main national and international guidelines [18–20]. Quinine is now relegated to a second-line treatment for cases where contraindications exist, or because of the unavailability of ACT or atovaquone–proguanil; likewise in complicated malaria treatment given within the first trimester of pregnancy due to the possibility of teratogenicity [7, 18–20].

Most studies concerning imported malaria have been carried out on patients with complicated malaria [11, 12] with artesunate being the most-commonly used drug for intravenous treatment. Besides producing faster parasite clearance and ending fever, it has a better safety profile (posing predominantly less risk of severe hypoglycaemia), shortens hospitalization and has recently been shown to reduce the length of time in ICU, above all in patients with more elevated parasitaemia [12]. No reduction in mortality has been demonstrated given the few patients who died in the studies thus making such analysis impossible, although with the solid evidence obtained in endemic regions, it would be unethical to carry out clinical trials with this objective in mind.

In the case of uncomplicated malaria caused by *P. falciparum*, the general recommendation is hospital admittance and oral treatment for at least 24–48 h [20], with the aim of observing the clinical evolution and tolerance to the drugs. Very few studies have been carried out with ACT in non-endemic zones. Hatz et al. [21] concluded that treatment with artemether–lumefantrine is safe and efficacious in those patients. A prospective observational multicentre study (MALTHER) compared the various treatment regimens for imported uncomplicated malaria in Europe [17]. There were a total of 18 different regimens although the most used were atovaquone–proguanil, quinine and artemether–lumefantrine. Compared to other regimens, quinine was associated with the highest non-completion rate, more secondary effects and the most days of hospitalization. In contrast, the artemether–mefloquine combination was linked to the fastest parasite clearance and cessation of fever. Regarding the period of hospitalization, the use of quinine was associated with longer hospital stays. In this study, unlike the MALTHER study, dihydroartemisinin–piperaquine was used as the ACT, the only drug of its type commercially available in Spain and presumably the main drug used in the majority of Spanish hospitals; this combination has also been demonstrated to be efficacious and safe both in endemic countries as well as in Europe [16, 22]. In addition, as belonging to just one centre, the two groups of patients compared were much more homogenous thus avoiding, to a large extent, any bias derived from variations in clinical practice between healthcare sites or in different countries.

The overall average hospital stay for patients in our study was 2.88 days. The fact that most of the patients are immigrants has probably contributed to a slightly longer stay to that recommended. The language barrier that exists with many of these patients, as well as the problems concerning frequent follow-up visits to the healthcare centres, often for economic or work-related reasons, means that it is common their doctors feel more comfortable keeping them admitted to hospital until they are sure of good treatment tolerance and response. The rapid parasite clearance and the markedly shorter treatment regimen using ACT are probably the factors that most influence the length of stay.

Atovaquone–proguanil is another medication recommended as a first-line treatment for uncomplicated malaria from *P. falciparum* [18–20]; however, the parasite clearance rate is longer than with ACT and the adverse effects, above all gastrointestinal, are similarly more frequent [17]. Furthermore, ACT has a very good profile in relation to possible *Plasmodium* resistance.

With regard to possible adverse reactions, the compounds derived from artemisinin have shown good tolerance and have a good safety profile [23, 24]. The most frequent adverse effects are: Type 1 hypersensitivity at the cutaneous level; a long QTc interval in the ECG; and, at the gastrointestinal level, nausea, vomiting and diarrhoea, although often this can be clinically confused with the malaria itself [25]. Cases have also been described of raised transaminase levels or hepatitis. This was only observed in one patient; and although there were no important consequences and the condition ceased after only a few days, it did result in more days spent in hospital.

The most severe adverse effect of artemisinin derivatives however is delayed haemolytic anaemia [25–28]. This is produced by the “pitting” mechanism, in which the parasitized erythrocytes, after being cleared of parasites by the spleen, are resealed and returned to the blood stream, but with a reduced lifespan of 1–3 weeks [28]. This lysis process occurs on average 2 weeks after the drug is administered, and in some cases can require a blood transfusion. Although this has mainly been seen in patients with complicated malaria (with elevated parasitaemia) treated with artesunate, it has also been described in patients treated with intramuscular artemether and oral artemether–lumefantrine [27]. As far as it is known, no case has been reported in relation to dihydroartemisinin–piperaquine. In this study, the Hb levels were higher even in the group treated with ACT at 7 and 28 days. This is probably because in patients with uncomplicated malaria, delayed haemolysis is the exception; in fact, the more rapidly the parasitaemia disappears and the ACT-treated patients recover, the more rapid the accompanying recovery of the basal hemoglobin levels as well.

With regard to pregnancy, there is insufficient information about the safety and efficacy of many anti-malarials, especially in the first trimester. Consequently, the only drugs considered safe are quinine, chloroquine, clindamycin and proguanil. Artemisinin-derived drugs are safe and efficacious in the second and third trimesters, in that they have been demonstrated not to be teratogenic [29, 30].

The limitations present in this study come from the fact that it is a retrospective work.

Furthermore, given the characteristics of the population that is the object of the study, the results may only be strictly extrapolated to African immigrants treated with two treatment regimens, one which has already passed to second-line use (quinine sulfate–doxycycline) and the other, an ACT (dihydroartemisinin–piperaquine), which is a first-line treatment. New prospective studies will need to be carried out to evaluate if any differences exist in terms of safety, efficacy and healthcare costs between the ACT-based treatments and, for example, atovaquone–proguanil, which is also a fixed combination over the same period (3 days in total). Moreover, with atovaquone–proguanil, ample experience has been gathered in non-endemic regions and it has a proven safety record.

## Conclusions

The results of this work reinforce the results of the few studies published to date regarding ACT treatments for patients with uncomplicated malaria in non-endemic regions. They are drugs with a good safety record, clearing parasitaemia more rapidly than other anti-malarials, and are probably more cost effective by reducing the average length of stay in hospital. Nonetheless, despite being safe drugs, one should be alert to the possibility of adverse effects such as hepatitis and delayed haemolytic anaemia.

## Additional file

10.1186/s12936-016-1408-1 General data table.

## Abbreviations

ACT: artemisinin-based combination therapy
Hb: haemoglobin
HBV: hepatitis B virus
HCV: hepatitis C virus
HIV: human immunodeficiency virus
ICU: intensive care unit
IQR: interquartile rate
PCR: polymerase chain reaction
VFR: visiting friends and relatives
