# Supplementary material for: Impact of using artemisinin-based combination therapy (ACT) in the treatment of uncomplicated malaria from Plasmodium falciparum in a non-endemic zone
Source: Malar J. 2016 Jul 2;15:339. doi: 10.1186/s12936-016-1408-1 (PMC4930579; doi:10.1186/s12936-016-1408-1)
Supplement: Supplementary file 1 — 10.1186/s12936-016-1408-1 General data table. [file 12936_2016_1408_MOESM1_ESM.docx]

| Gender | Year | Age  (years) | Type of  traveller | Hours  of  hospitalization. | Group of treatment | Parasitemia | Time in which  parasitemia  becomes  negative.  Hours | Country  of origin | Hb  (g/dL)  Day 1 | Hb  (g/dL)  Day 7 | Hb  (g/dL)  Day 28 | Platelets  x10^3^/µL |
| --- | --- | --- | --- | --- | --- | --- | --- | --- | --- | --- | --- | --- |
| Male | 2011 | 31 | VFR | 138 | Quinine/atovaquone-proguanil | <1% |  | Mali | 15,1 | 12,6 |  | 56 |
| Male | 2011 | 34 | VFR | 64 | Quinine/atovaquone-proguanil | <1% |  | Mali | 13,2 | 12,6 |  | 111 |
| Male | 2011 | 30 | VFR | 92 | Quinine/atovaquone-proguanil | <1% | 24 | Mali | 13 | 12,1 | 14,6 | 92 |
| Male | 2011 | 29 | VFR | 141 | Quinine/atovaquone-proguanil | <1% |  | Mali | 12 | 12,8 | 13,2 | 37 |
| Male | 2011 | 31 | VFR | 112 | Quinine/atovaquone-proguanil | 1-2% | 72 | Mali | 11,5 | 11,1 | 14,2 | 79 |
| Male | 2011 | 29 | VFR | 68 | Quinine/atovaquone-proguanil | >2% y 5% | 48 | Mali | 12,2 | 12,9 |  | 53 |
| Male | 2011 | 33 | VFR | 87 | Quinine/atovaquone-proguanil | >2% y 5% | 24 | Mali | 13,9 | 13,2 | 13,3 | 116 |
| Male | 2012 | 30 | VFR | 126 | Quinine/atovaquone-proguanil | <1% | 24 | Mali | 15 | 12,9 | 13,1 | 38 |
| Male | 2012 | 28 | VFR | 87 | Quinine/atovaquone-proguanil | 1-2% |  | Mali | 14,2 | 11,2 | 13,8 | 120 |
| Male | 2012 | 32 | VFR | 112 | Quinine/atovaquone-proguanil | 1-2% | 48 | Mali | 14 | 13,8 | 14,2 | 105 |
| Male | 2012 | 36 | VFR | 140 | Quinine/atovaquone-proguanil | >2% y 5% | 72 | Mali | 11,8 | 10,6 | 10,2 | 70 |
| Male | 2012 | 30 | VFR | 39 | Dihydroartemisinin-piperaquine | 1-2% | 48 | Mali | 12,2 | 11,7 | 12,4 | 118 |
| Male | 2012 | 33 | VFR | 67 | Dihydroartemisinin-piperaquine | 1-2% | 24 | Mali | 12,5 | 13,4 | 13,7 | 121 |
| Male | 2013 | 32 | VFR | 43 | Dihydroartemisinin-piperaquine | <1% | 24 | Mali | 12,6 | 13,3 | 13,7 | 101 |
| Male | 2013 | 29 | VFR | 43 | Dihydroartemisinin-piperaquine | <1% | 48 | Mali | 12,7 |  |  | 183 |
| Male | 2013 | 30 | VFR | 91 | Dihydroartemisinin-piperaquine | <1% | 24 | Mali | 15,6 | 14,8 | 16,1 | 73 |
| Male | 2013 | 27 | VFR | 46 | Dihydroartemisinin-piperaquine | <1% | 24 | Mali | 14,3 |  |  | 113 |
| Male | 2013 | 42 | VFR | 72 | Dihydroartemisinin-piperaquine | <1% | 48 | Mali | 12,9 | 12,3 |  | 69 |
| Male | 2013 | 42 | VFR | 69 | Dihydroartemisinin-piperaquine | <1% | 24 | Mali | 12,7 | 12,9 | 14,2 | 116 |
| Male | 2013 | 31 | VFR | 68 | Dihydroartemisinin-piperaquine | 1-2% | 72 | Mali | 14,3 | 13,6 |  | 95 |
| Male | 2013 | 30 | VFR | 73 | Dihydroartemisinin-piperaquine | 1-2% | 24 | Mali | 15,3 | 16 |  | 77 |
| Male | 2013 | 24 | VFR | 93 | Dihydroartemisinin-piperaquine | 1-2% | 24 | Mali | 15,5 | 15,8 | 15,2 | 142 |
| Male | 2013 | 22 | VFR | 92 | Dihydroartemisinin-piperaquine | 1-2% | 48 | Mali | 14,4 | 13,3 |  | 86 |
| Male | 2013 | 47 | VFR | 49 | Dihydroartemisinin-piperaquine | 1-2% | 24 | Mali | 14,6 | 12,1 | 12,7 | 75 |
| Male | 2013 | 28 | VFR | 69 | Dihydroartemisinin-piperaquine | 1-2% | 24 | Mali | 13,8 | 12,3 |  | 130 |
| Male | 2013 | 36 | VFR | 135 | Dihydroartemisinin-piperaquine | >2% y 5% | 48 | Mali | 14 | 11,9 | 12,6 | 23 |
| Male | 2014 | 30 | VFR | 67 | Dihydroartemisinin-piperaquine | <1% | 24 | Mali | 11,8 | 14,7 |  | 173 |
| Male | 2014 | 34 | VFR | 67 | Dihydroartemisinin-piperaquine | <1% | 24 | Mali | 12,6 | 13,4 |  | 62 |
| Male | 2014 | 32 | VFR | 48 | Dihydroartemisinin-piperaquine | <1% | 24 | Mali | 15,4 | 16 |  | 95 |
| Male | 2014 | 35 | VFR | 49 | Dihydroartemisinin-piperaquine | <1% |  | Mali | 10,1 | 11 |  | 153 |
| Male | 2014 | 44 | VFR | 64 | Dihydroartemisinin-piperaquine | 1-2% | 48 | Mali | 13,2 | 12,2 | 13,4 | 35 |
| Male | 2014 | 28 | VFR | 70 | Dihydroartemisinin-piperaquine | 1-2% | 24 | Mali | 14,2 | 14,7 | 14,4 | 61 |
| Male | 2015 | 25 | VFR | 36 | Dihydroartemisinin-piperaquine | <1% | 24 | Mali | 12 | 12 | 13,4 | 197 |
| Male | 2015 | 34 | VFR | 45 | Dihydroartemisinin-piperaquine | <1% | 24 | Mali | 13,8 |  |  | 67 |
| Male | 2015 | 35 | VFR | 71 | Dihydroartemisinin-piperaquine | <1% | 24 | Mali | 12,5 | 12,6 | 13,9 | 47 |
| Male | 2015 | 51 | VFR | 24 | Dihydroartemisinin-piperaquine | <1% | 24 | Mali | 12,8 | 13,9 | 15,8 | 235 |
| Male | 2015 | 32 | VFR | 44 | Dihydroartemisinin-piperaquine | <1% | 24 | Mali | 14,5 | 12,9 | 14,2 | 67 |
| Male | 2015 | 38 | VFR | 64 | Dihydroartemisinin-piperaquine | <1% | 24 | Mali | 15,1 | 13 | 15 | 58 |
| Male | 2015 | 31 | VFR | 63 | Dihydroartemisinin-piperaquine | <1% | 24 | Mali | 13,9 | 14,5 | 14,5 | 160 |
| Male | 2015 | 34 | VFR | 45 | Dihydroartemisinin-piperaquine | <1% | 24 | Mali | 13,8 | 13,1 |  | 67 |
| Male | 2015 | 34 | VFR | 65 | Dihydroartemisinin-piperaquine | <1% | 24 | Mali | 14,8 | 14,4 |  | 106 |
| Male | 2015 | 38 | VFR | 60 | Dihydroartemisinin-piperaquine | 1-2% | 24 | Mali | 14,7 | 12,3 | 15,7 | 42 |
| Male | 2015 | 28 | VFR | 25 | Dihydroartemisinin-piperaquine | 1-2% | 48 | Mali | 14 | 14 |  | 339 |
| Male | 2015 | 41 | VFR | 44 | Dihydroartemisinin-piperaquine | >2% y 5% | 48 | Mali | 13,2 | 13,3 | 14,5 | 97 |
| Male | 2006 | 28 | VFR | 112 | Quinine/atovaquone-proguanil | >2% y 5% | | Ghana | 15,8 | 13,7 |  | 78 |
| Male | 2007 | 46 | VFR | 183 | Quinine/atovaquone-proguanil | 1-2% |  | Ghana | 15,4 | 14,1 | 14,4 | 71 |
| Male | 2007 | 27 | VFR | 88 | Quinine/atovaquone-proguanil | >2% y 5% | 72 | Ghana | 14,3 | 12,6 |  | 37 |
| Male | 2010 | 28 | VFR | 70 | Quinine/atovaquone-proguanil | 1-2% | 24 | Ghana | 13,8 | 11,9 | 12,6 | 107 |
| Male | 2010 | 17 | VFR | 120 | Quinine/atovaquone-proguanil | >2% y 5% | | Ghana | 10,1 | 11,9 | 13,4 | 63 |
| Male | 2011 | 32 | VFR | 45 | Quinine/atovaquone-proguanil | <1% |  | Ghana | 14 | 13,6 | 14,2 | 100 |
| Male | 2011 | 33 | VFR | 142 | Quinine/atovaquone-proguanil | <1% |  | Ghana | 12,6 | 10,5 |  | 51 |
| Male | 2012 | 42 | VFR | 141 | Quinine/atovaquone-proguanil | <1% |  | Ghana | 14,9 | 15,5 |  | 176 |
| Male | 2011 | 33 | VFR | 96 | Quinine/atovaquone-proguanil | <1% |  | Ghana | 13,2 | 13,6 | 13,6 | 151 |
| Male | 2015 | 16 | VFR | 69 | Dihydroartemisinin-piperaquine | <1% | 24 | Ghana | 11,8 | 13,3 |  | 114 |
| Female | 2015 | 39 | VFR | 21 | Dihydroartemisinin-piperaquine | <1% | 24 | Ghana | 10,3 | 10 |  | 75 |
| Male | 2015 | 41 | VFR | 67 | Dihydroartemisinin-piperaquine | <1% | 24 | Ghana | 12,2 | 11,5 | 11,7 | 166 |
| Male | 2015 | 26 | VFR | 64 | Dihydroartemisinin-piperaquine | <1% | 24 | Ghana | 14,7 | 12,8 | 14,3 | 49 |
| Male | 2004 | 34 | VFR | 94 | Quinine/atovaquone-proguanil | 1-2% |  | Guinea Bissau | 15 | 12,9 |  | 94 |
| Male | 2010 | 47 | VFR | 98 | Quinine/atovaquone-proguanil | <1% |  | Guinea Bissau | 11,7 | 10 | 12,9 | 100 |
| Male | 2011 | 68 | VFR | 141 | Quinine/atovaquone-proguanil | <1% | 48 | Guinea Bissau | 13,7 | 12,5 | 13,6 | 61 |
| Male | 2012 | 38 | No | 141 | Dihydroartemisinin-piperaquine | <1% | 24 | Guinea Bissau | 16,2 | 15,1 | 16,8 | 55 |
| Male | 2015 | 58 | VFR | 45 | Dihydroartemisinin-piperaquine | <1% | 24 | Guinea Bissau | 13,6 |  |  | 201 |
| Male | 2005 | 37 | VFR | 189 | Quinine/atovaquone-proguanil | >2% y 5% | | Senegal | 13,3 | 10,8 | 12,7 | 167 |
| Male | 2007 | 23 | VFR | 139 | Quinine/atovaquone-proguanil | <1% |  | Senegal | 16,9 | 12,8 | 14,1 | 168 |
| Male | 2010 | 36 | No | 49 | Quinine/atovaquone-proguanil | <1% | 72 | Senegal | 15,1 | 15,1 | 14,7 | 306 |
| Male | 2010 | 21 | VFR | 64 | Quinine/atovaquone-proguanil | <1% | 48 | Senegal | 14,1 | 13,6 | 13,6 | 172 |
| Male | 2010 | 31 | VFR | 89 | Quinine/atovaquone-proguanil | <1% | 24 | Senegal | 14,4 | 13,8 | 14,8 | 30 |
| Male | 2011 | 21 | No | 94 | Quinine/atovaquone-proguanil | <1% |  | Senegal | 13,6 | 14,6 | 14,5 | 127 |
| Male | 2012 | 21 | VFR | 43 | Dihydroartemisinin-piperaquine | <1% | 24 | Senegal | 15,4 | 15,8 |  | 207 |
| Male | 2012 | 24 | VFR | 45 | Dihydroartemisinin-piperaquine | 1-2% | 48 | Senegal | 14 | 12,1 | 13,9 | 114 |
| Male | 2013 | 45 | VFR | 64 | Dihydroartemisinin-piperaquine | <1% | 24 | Senegal | 12,2 | 11,7 | 14,3 | 90 |
| Male | 2013 | 43 | VFR | 51 | Dihydroartemisinin-piperaquine | <1% | 24 | Senegal | 14,7 | 14,7 | 15,4 | 51 |
| Male | 2013 | 39 | VFR | 114 | Dihydroartemisinin-piperaquine | 1-2% | 24 | Senegal | 12,3 | 10,5 | 12,7 | 40 |
| Male | 2013 | 37 | VFR | 69 | Dihydroartemisinin-piperaquine | >2% y 5% | 24 | Senegal | 14,5 | 12,6 |  | 85 |
| Male | 2014 | 33 | VFR | 63 | Dihydroartemisinin-piperaquine | 1-2% | 48 | Senegal | 14,5 | 14,5 | 14,9 | 32 |
| Male | 2013 | 26 | VFR | 53 | Dihydroartemisinin-piperaquine | <1% | 24 | Senegal | 14,2 | 15,8 | 14,3 | 111 |
| Female | 2009 | 53 | No | 69 | Quinine/atovaquone-proguanil | <1% |  | Equatorial Guinea | 10,8 |  | 9,1 | 295 |
| Female | 2011 | 22 | VFR | 69 | Quinine/atovaquone-proguanil | 1-2% | 96 | Equatorial Guinea | 11,1 | 10 |  | 103 |
| Male | 2004 | 26 | VFR | 39 | Quinine/atovaquone-proguanil | 1-2% |  | Nigeria | 13,5 |  |  | 84 |
| Male | 2013 | 33 | VFR | 141 | Dihydroartemisinin-piperaquine | >2% y 5% | 48 | Nigeria | 15,5 | 14,2 |  | 61 |
| Male | 2012 | 36 | VFR | 69 | Quinine/atovaquone-proguanil | 1-2% | 24 | Burkina Fasso | 12,4 | 13,9 | 14,6 | 88 |
| Male | 2011 | 56 | VFR | 117 | Quinine/atovaquone-proguanil | <1% | 24 | Gambia | 13,3 | 11,5 |  | 46 |
| Male | 2013 | 28 | VFR | 93 | Dihydroartemisinin-piperaquine | 1-2% | 24 | Gambia | 16,4 | 14,8 | 16,2 | 33 |
| Male | 2014 | 26 | Others | 162 | Dihydroartemisinin-piperaquine | <1% | 24 | Spain | 16,3 | 14,4 | 15,1 | 146 |
| Male | 2012 | 31 | VFR | 87 | Dihydroartemisinin-piperaquine | 1-2% | 48 | Ivory Coast | 15,1 | 14,3 | 14,6 | 177 |
